# Metastatic testicular retroperitoneal teratoma in an adult: Case report

**DOI:** 10.1016/j.ijscr.2019.06.004

**Published:** 2019-06-12

**Authors:** Hanan M. Alghamdi

**Affiliations:** a King Fahad Hospital of the University, Imam Abdulrahman Bin Faisal University College of Medicine, Department of Surgery, Saudi Arabia; b Almaan General Hospital, Saudi Arabia

**Keywords:** Teratoma, Testicular tumors, Retroperitoneal mass, Germ cell tumor, Ovarian tumors, Sarcomas

## Abstract

**Introduction:**

Teratomas are bizarre neoplasms derived from embryonic tissues typically found in the gonads and sacrococcygeal tissues of adults.

**Case presentation:**

We report a case of histologically proven metastatic teratoma with an unusual presentation. The 32-year old male patient had a retroperitoneal tumour detected on computed tomography during the workup of abdominal pain.

**Discussion:**

Retroperitoneal teratomas are uncommon and a challenge to manage. Differential diagnoses could be a dilemma without a thorough history and a high index of suspicion.

**Conclusions:**

Recurrent metastatic teratoma is one of the rare differential diagnoses of any retroperitoneal mass and can present years after complete resection of the primary tumour.

## Highlights

• Differential diagnoses of retroperitoneal masses should remain wide in adults.
• Teratomas should be considered as a potential cause of abdominal pain and distention in young adults.
• Testicular examination and past history of any intervention should be sought inn any retroperitoneal masses.

## 1 Introduction

Teratomas are originally somatic cells derived from two or more germ layers (ectoderm, mesoderm, and/or endoderm). Typically, teratomas are non-seminomatous germ cell tumours located in either the sacrococcygeal region or in the gonads. Although the paediatric age group is most affected, adults can also develop teratomas in various anatomical locations. These include the gonads predominantly, but also in areas such as the sacrococcix retroperitoneum and mediastinum. Retroperitoneal teratomas in adults are rare and represent only 1–11% of all primary teratomas. Teratomas are generally considered to be benign in the first stages and patients are generally asymptomatic. Malignant mature cystic teratomas (0.2–2% of cases) [1] however, have the potential to metastasize to sites such as the retroperitoneal lymph nodes and lung [2]. Symptoms typically arise as a result of teratoma size, and patients frequently present with a palpable mass and abdominal distension. Diagnosis can be established and the solid and/or cystic components demonstrated by ultrasound. Computerized tomography and magnetic resonance imaging, both superior to ultrasound, are used to establish the extent of the tumour in relation to adjacent organs. Occasionally angiography is used to detect and assess the blood supply. This work has been reported in line with the SCARE criteria [3].

## 2 Case presentation

**Fig. 1:**
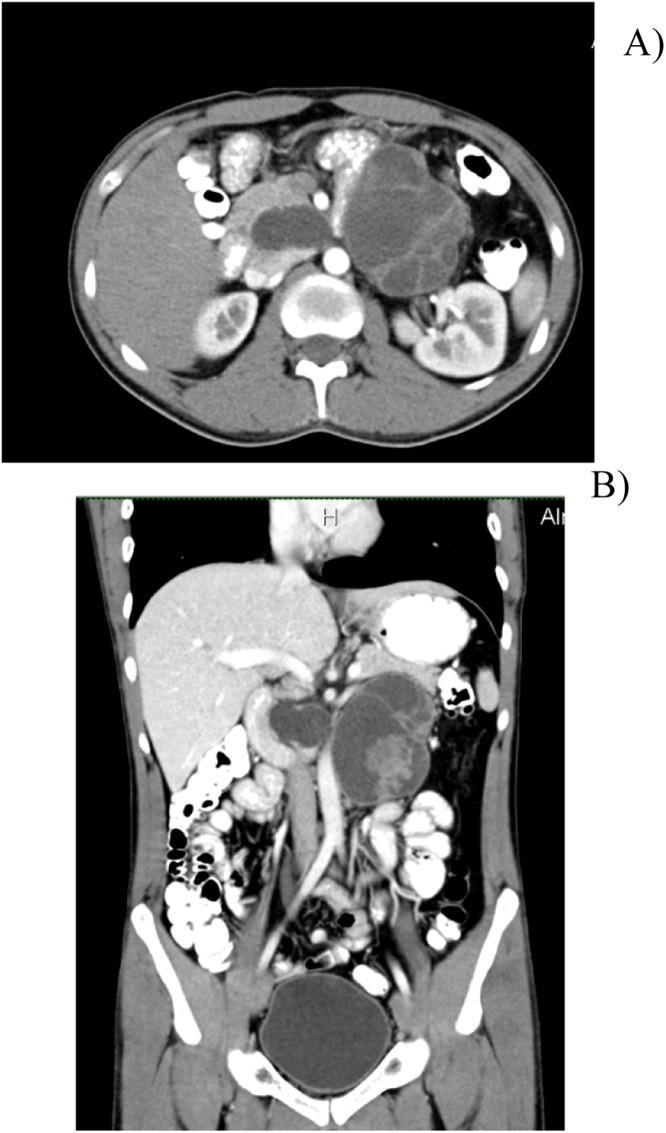
A) Abdominopelvic CT scan with I/V contrast report showing 2 lesions in the pancreas. B) Coronal section demonstrates the cystic lesion with IV contrast.

**Fig. 2:**
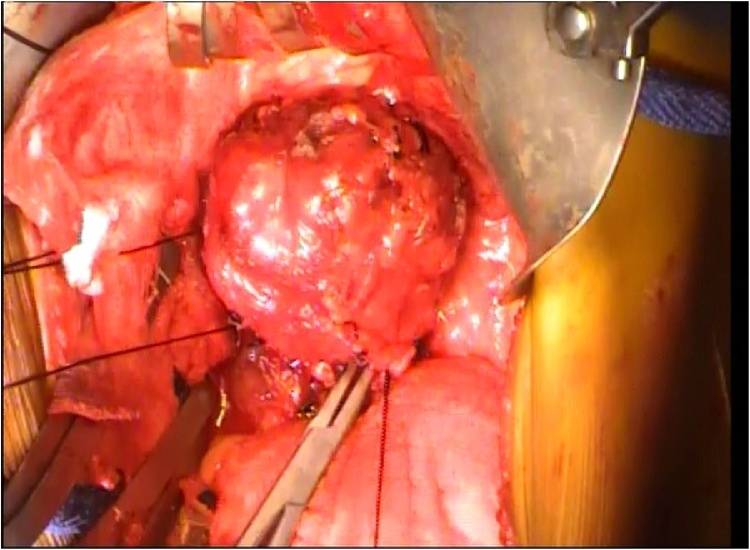
Intraoperative picture demonstrates the teratoma.

**Fig. 3:**
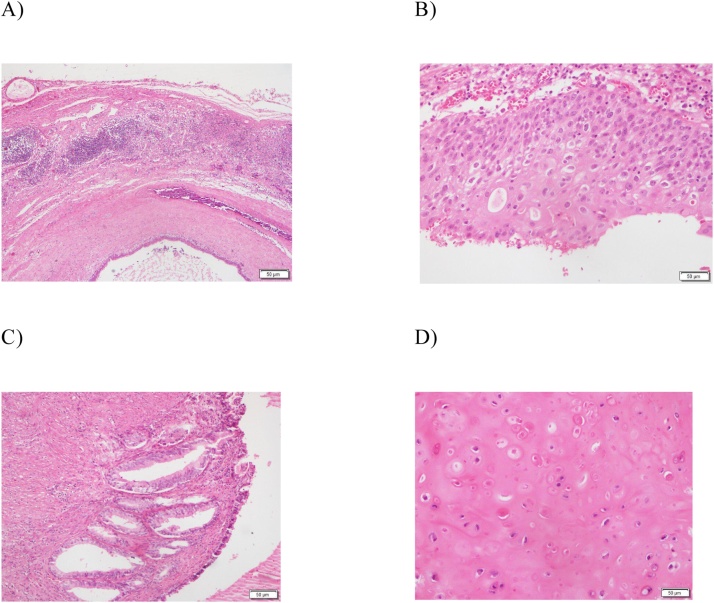
Histopathology showing mixed embryonic cell. A) Lymph node with metastatic cystic neoplasm. B) Cyst lining is formed of nonkeratinized squamous epithelium. C) Cyst also entangling glandular and smooth muscle elements. D) Areas of hyaline cartilage.

**Fig. 4:**
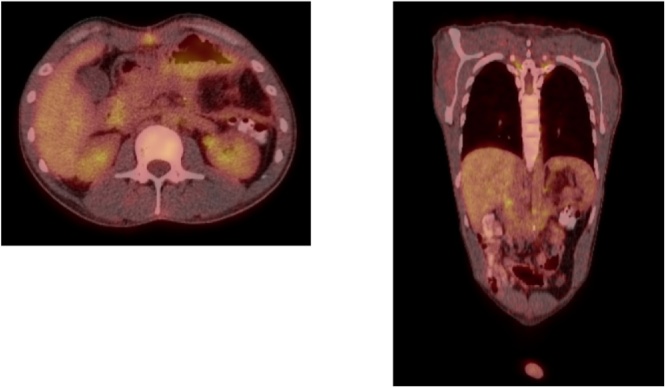
PET scan showing no recurrence in the surgical bed or other lesion 3 months after surgery.

A 31-years old male patient with no medical comorbidity presented to our facility. Six years prior to this presentation he had an isolated left testicular mass detected on physical examination and CT which proved to be a malignant germ cell tumour. He underwent left orchiectomy but retroperitoneal lymph node dissection (RPLND) was not carried out as the CT showed no enlarged lymph nodes and histopathology showed the presence of a teratoma and yolk sac tumour. No further details however were mentioned. Accordingly, he received four cycles of chemotherapy (Cisplatin, Bleomycin, and Etoposide). He did well for six years, had no major complaints, and underwent only one follow up ultrasound which showed no suspicious abdominal swellings. He presented to us with on and off upper abdominal pain of six months duration, with no other associated symptoms. On physical examination he was vitally stable with a soft and lax abdomen but mild tenderness in the upper central part of the abdomen and a deep mass. Groin examination revealed signs of a left orchiectomy; the right testicle was in normal position with no swelling or other abnormality detected. Abdominopelvic US showed a large retroperitoneal semisolid mass measuring 8 by 6 cm, abating the body and tail of the pancreas. Chest and abdominopelvic CT scan with IV and oral contrast were carried out and showed two lesions, one abating the head of the pancreas (3 × 2 cm in size) and the other baiting the body and tail of the pancreas with clear separation distal (Fig. 1). All tumour markers, including B-HCG, AFP, CA 19-9, CEA, and CA 19-9, were within the normal range. The patient underwent midline laparotomy and complete resection both retroperitoneal masses with preservation of the pancreas (Fig. 2). The recovery was uneventful, and the patient was discharged five days postoperatively. Histopathology of the masses showed a metastatic germ cell tumour of the teratoma component in the background of lymphoid tissue (Fig. 3). Thus, the tumour board diagnosed this as stage IV metastatic germ cell tumour of the teratoma, and recommended to commence the patient on chemotherapy (Cisplatin, Bleomycin, and Etoposide). The patient tolerated four cycles of chemotherapy. The follow-up PET scan at six months postoperatively showed no trace or recurrence of the tumour (Fig. 4). Currently, the patient has been followed up with ultrasound for two years with no recurrence.

## 3 Discussion

Teratomas are considered the most common type of germ cell tumour in humans; most of these neoplasms are benign. Teratomas are typically classified into three general categories: mature (cystic/solid and benign), immature (malignant), or mondermal (highly specialized) [1]. Each of these histological types may present alone or in combination with others. A mature teratoma consists of an adult-type tumour with well-differentiated elements, while an immature teratoma consists of elements with only partial somatic differentiation, resembling those seen in embryonic or foetal tissue [4]. Teratomas often contain dermal elements and can be classified by the type of epithelium and dermal tissue contained within the tumour. Epidermoid teratomas contain stratified squamous epithelium, dermoid teratomas may contain any kind of epithelium and dermal elements such as hair and glands, and teratoid teratomas are lined with any respiratory epithelium type (usually columnar epithelium) and contain sebum [2].

In general, teratomas are considered foreign to the anatomical region in which they are found despite their tendency to differentiate into somatic tissue types. It has been proposed that these cysts may develop from meiosis I failure or from a premeiotic cell in which meiosis I has failed [1]. Retroperitoneal teratomas in children comprise only 3.5–4% of all germ cell tumours and arise from germ cells that fail to mature normally in the gonads. These totipotent cells can differentiate into tissue components representing derivatives of mesoderm, ectoderm, and endoderm [2]. The anatomical distribution of teratomas are described in order of decreasing frequency: ovaries, testes, anterior mediastinum, retroperitoneal space, presacral and coccygeal areas, pineal and other intracranial sites, abdominal viscera other than the gonads, and neck [1].

Retroperitoneal teratomas are rare entities, representing only 1%–11% of all primary retroperitoneal tumours. Incidence is bimodal with peaks in the first six months of life and in early adulthood. Due to their location, retroperitoneal teratomas are usually identified only after they have grown to a substantial size [5,6]. Additionally, the incidence of retroperitoneal teratomas in females is twice that seen in males. When retroperitoneal teratomas do occur, they are often located near the upper pole of the kidney with left-sided preponderance. Although these tumours are mostly asymptomatic, they can cause abdominal distension and mass effect (pain, nausea, and vomiting) via compression of surrounding structures [5]. Apart from teratomas, the differential diagnoses of retroperitoneal masses include lymph nodes, ovarian tumours, renal cysts, adrenal tumours, retroperitoneal fibromas, sarcomas, haemangiomas, xantogranuloma, and perirenal abscess [7]. There is no specific tumour marker for teratomas; however, immature (malignant) teratomas have been associated with elevated AFP (alpha-fetoprotein) levels [8].

The diagnosis of retroperitoneal teratoma can often be made on the basis of imaging. These teratomas are predominantly cystic but can be solid in appearance. Computed tomography (CT) or magnetic resonance imaging (MRI) can identify various components of these tumours, including bone, soft tissue density structures, adipose tissue, and sebaceous and serous-type fluids. These imaging studies can also display the precise location and morphology of and adjacent structures to the tumour, which allow for better preoperative mapping [4]. Testicular ultrasound is necessary to rule out a coexisting testicular germ cell tumour in male patients. This is essential as 50% of men with retroperitoneal tumours also have testicular carcinoma in situ, a precursor for testicular germ cell tumours [8].

Surgical resection remains the mainstay of therapy for mature teratomas and is required for a definitive diagnosis [6]. Resectability is determined by the pathological category and extent of the tumour. Resection can include segments of the GI tract, kidney, bladder, spleen, aorta, and vena cava [9]. Torsion is common with these tumours, and if rupture occurs, sebaceous material can spill out into the abdominal cavity, causing shock, haemorrhage, or a marked granulomatous reaction that can lead to the formation of adhesions [1]. Resection of benign tumours results in an almost 100% five-year survival rate. In long-term studies the best survival rates for primary retroperitoneal tumours and disease-free survival is associated with complete surgical resection [9]. Furthermore, the risk of malignant transformation of teratoma to carcinoma or sarcoma is well documented. Therefore, unresected teratoma may result in late relapse (defined as recurrence after a relapse-free interval of more than two years following completion of primary treatment). A late relapse often shows slow growth and is associated with poor response to chemotherapy. Long-term care also involves advising the patient to have an annual CT scan in order to detect relapse at an asymptomatic phase [10,11]. Our case represents the unusual histopathological finding of a retroperitoneal mass in an adult male patient years after complete resection of a retroperitoneal teratoma but with non-compliance in follow up. This case may represent a long-term distant recurrence of the primary testicular tumour, perhaps due to surgical technique used before rather than a metachronous form of a gonadal germ cell tumour.

## 4 Conclusion

Primary retroperitoneal teratoma is a rare entity in adults. Although usually asymptomatic, large neoplasms can cause abdominal and flank pain. Preoperatively, the diagnosis can be established by its characteristic appearance on computer tomography. The definitive treatment for these neoplasms is surgical resection. The differential diagnosis of such a mass should include unusual pathology. Moreover, teratoma can present as primary or distant metastases and long-term follow up is warranted.

## Declaration of Competing Interest

None.

## Funding

This research did not receive any specific grant from funding agencies in the public, commercial, or not-for-profit sectors.

## Ethical approval

Case reports are exempted from ethical approval according to policies of Imam Abdulrahman Bin Faisal University.

## Consent

Written informed consent was obtained from the patient for publication of this case report.

## Author contribution

Hanan AlGhamdi: performed the operation, study concept, study design, data collection, writing the paper, reviewing and editing the final manuscript.

## Registration of research studies

N/A.

## Guarantor

Dr. Hanan Alghamdi.

## Provenance and peer review

Not commissioned, externally peer-reviewed.
